# Multiple-image super-resolution of cryo-electron micrographs based on deep internal learning

**DOI:** 10.1017/S2633903X2300003X

**Published:** 2023-02-09

**Authors:** Qinwen Huang, Ye Zhou, Hsuan-Fu Liu, Alberto Bartesaghi

**Affiliations:** 1 Department of Computer Science, Duke University, Durham, North Carolina, USA; 2 Department of Biochemistry, Duke University School of Medicine, Durham, North Carolina, USA; 3 Department of Electrical and Computer Engineering, Duke University, Durham, North Carolina, USA

**Keywords:** Cryo-electron microscopy, image super-resolution, single-particle analysis, zero-shot learning

## Abstract

Single-particle cryo-electron microscopy (cryo-EM) is a powerful imaging modality capable of visualizing proteins and macromolecular complexes at near-atomic resolution. The low electron-doses used to prevent radiation damage to the biological samples, however, result in images where the power of the noise is 100 times greater than the power of the signal. To overcome these low signal-to-noise ratios (SNRs), hundreds of thousands of particle projections are averaged to determine the three-dimensional structure of the molecule of interest. The sampling requirements of high-resolution imaging impose limitations on the pixel sizes that can be used for acquisition, limiting the size of the field of view and requiring data collection sessions of several days to accumulate sufficient numbers of particles. Meanwhile, recent image super-resolution (SR) techniques based on neural networks have shown state-of-the-art performance on natural images. Building on these advances, here, we present a multiple-image SR algorithm based on deep internal learning designed specifically to work under low-SNR conditions. Our approach leverages the internal image statistics of cryo-EM movies and does not require training on ground-truth data. When applied to single-particle datasets of apoferritin and T20S proteasome, we show that the resolution of the 3D structure obtained from SR micrographs can surpass the limits imposed by the imaging system. Our results indicate that the combination of low magnification imaging with in silico image SR has the potential to accelerate cryo-EM data collection by virtue of including more particles in each exposure and doing so without sacrificing resolution.

## Impact Statement

This research paper describes an image super-resolution method that improves the quality of single-particle cryo-EM images and results in higher resolution reconstructions of protein structures. By leveraging internal image statistics of cryo-EM movies, we propose to use a deep-learning framework that is self-supervised and does not require training on ground-truth images. This work is addressed to people working at the interface between biological imaging and computer vision. The proposed approach is validated on real single-particle cryo-EM datasets and we show that the resolution of 3D structures can surpass the limits imposed by the imaging system. This advance can potentially accelerate cryo-EM data collection and pave the way for improving the throughput of structure determination using single-particle cryo-EM.

## 1. Introduction

Single-particle cryo-electron microscopy (cryo-EM) is a powerful imaging modality used to determine the three-dimensional structure of proteins and macromolecular complexes at near-atomic resolution^(1–4)^. By combining hundreds of thousands of noisy projection images of identical copies of the molecule of interest taken from different orientations, 3D reconstructions can be obtained where molecular level details can be visualized. While the signal contributed by each individual projection is very weak, averaging the contribution from many particles allows to overcome the extremely low signal-to-noise ratios (SNRs). Acquiring such large datasets, however, is time consuming and can take several days to complete, becoming a bottleneck in the structure determination pipeline. One strategy to improve the throughput of data collection is to increase the size of the field of view by acquiring images at lower magnification. For example, doubling the pixel size will increase the imaging area fourfold, resulting in four times as many particles per exposure. While this strategy will in principle limit the attainable resolution due to the coarser spatial sampling, it does not mean a permanent loss of high-frequency information because images are collected in movie-mode and motion exists between the acquired frames. Super-resolution (SR) is a widely studied problem in the field of natural image photography. In recent years, the SR field has focused on single-image super-resolution (SISR) where a high-resolution (HR) image is obtained from a single low-resolution (LR) input. However, SISR is mostly limited to adding high-frequency information from learned image priors. On the other hand, multi-image super-resolution (MISR) aims to reconstruct the original HR signal using multiple LR images. When sub-pixel motion exists, each LR image provides different LR samples of the underlying higher-resolution scene. MISR approaches exploit this additional information to achieve the recovery of the HR signal. This same principle presents the opportunity of using MISR to overcome resolution constraints in single-particle cryo-EM.

**Figure 1.**
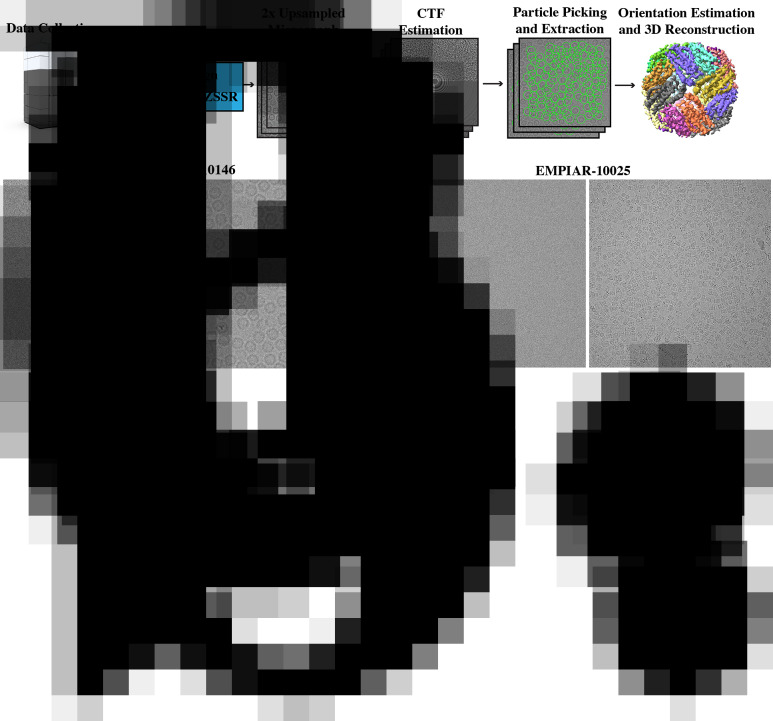
Super-resolution single-particle structure determination pipeline and example micrographs from two cryo-EM datasets. (a) Cryo-EM movies are collected using a large pixel size and subsequently upsampled by a factor of 2 using our self-supervised cryo-zero-shot super-resolution (cryo-ZSSR) approach. Super-resolved micrographs are then fed into the standard single-particle reconstruction workflow producing three-dimensional structures at resolutions surpassing the Nyquist rate. The 2× upsampling factor effectively results in a 4x speedup in the rate of data acquisition allowing the collection of four times more particles in the same amount of time. (b) Left: Example of a single raw frame from a movie of apoferritin from EMPIAR-10146 collected at 2 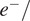 Å^2^. Right: Average of 50 frames corresponding to a total dose of 100 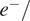 Å^2^. (c) Left: Example of a single raw frame from a movie of T20S proteasome from EMPIAR-10025 collected at 1.4 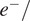 Å^2^. Right: Average of 38 frames corresponding to a total dose of 53.2 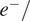 Å^2^.

Many SR algorithms based on machine learning have been proposed that achieve state-of-the-art (SotA) performance on natural images. Here, we set out to explore whether these strategies can be extended to work on low-SNR images such as the noisy projections obtained in single-particle cryo-EM. We propose to use a MISR algorithm that utilizes deep internal learning as initially presented in the zero-shot super-resolution (ZSSR) framework^(5)^ for SISR. Indeed, learning based on internal statistics has shown promising results for cryo-EM image denoising^(6,7)^. Internal data repetition at different frequency levels occurs naturally in single-particle cryo-EM: first, movie frames that are acquired earlier in the exposure contain more high-frequency signal than movie frames acquired later in the exposure; and second, each exposure area contains hundreds of naturally occurring projections of the same macromolecule. Our algorithm, cryo-zero-shot super-resolution (cryo-ZSSR), exploits cross scale internal data repetition in noisy movies obtained from frozen hydrated protein samples imaged under an electron microscope. We train a movie-specific neural network that takes in multiple frames from each LR movie and reconstructs a single 2× SR image per exposure area. These SR images are then fed into the standard cryo-EM data processing pipeline and used to generate a 3D reconstruction of the protein of interest (Figure 1). Our SR algorithm is self-supervised and does not require training on ground-truth HR data, which is nevertheless not available in cryo-EM. We evaluate the performance of our approach on two real datasets of apoferritin and T20S proteasome and show that the SR images can produce higher resolution reconstructions compared to the LR data. Used in combination with low-magnification imaging, our approach can be used to accelerate data collection while still producing high-quality 3D reconstructions.

## 2. Related Work

There has been a tremendous amount of work on image SR in the past couple of decades, both for SISR and for MISR. Traditional SISR algorithms can be divided into three categories: interpolation-based methods (e.g., bilinear, Lanczos kernels, etc.), reconstruction-based methods^(8,9)^, and example-based methods^(10,11)^. Interpolation-based algorithms are straightforward and fast but suffer from limited accuracy. Reconstruction-based methods make use of prior knowledge about images by restricting the possible solution space to generate high-quality images. However, these methods are usually time-consuming and their performance degrades rapidly as the upsampling factors increase. Example-based methods usually leverage machine learning to analyze relationships between the LR and its corresponding HR counterparts from training examples. More recently, deep-learning-based SISR algorithms^(12,13)^ built upon example-based learning received wide attention and demonstrated great superiority compared to more traditional approaches.^(12,13)^ Generative adversarial network (GAN)^(14)^ based deep-learning approaches, such as Super Resolution Generative Adversarial Network (SRGAN)^(15)^, and Photo Sampling via Latent Space Exploration (PULSE)^(16)^ are able to perform extremely well on certain natural and facial images. However, most of these networks are trained in a supervised manner and require knowledge of ground-truth images. Results reconstructed using GANs, even though visually appealing, tend to generate information that does not exist in the actual HR pictures. In addition, the formation of training datasets, specifically the LR images, are usually generated using predetermined ideal processes (e.g., bicubic downsampling, Gaussian blurring, etc.). In reality, LR images rarely follow this model, resulting in poor performance of previously mentioned SotA methods. To overcome this limitation, ZSSR was proposed by Shocher et al.^(5)^. Instead of relying on prior training, this method exploits the internal recurrence of information inside a single image and trains an image-specific CNN on examples extracted solely from the input image itself. Thus, ZSSR is able to achieve and outperform SotA methods on LR images generated under nonideal downsampling models.

Turning to the problem of MISR, which involves the extraction of information from many LR observations of the same scene to reconstruct HR images, the earliest method developed by Tsai and Huang^(17)^ used a frequency domain technique to improve the spatial resolution of images by combining multiple LR images with sub-pixel accuracy displacements. Later on, other spatial domain MISR methods were proposed that include nonuniform interpolation such as adaptive kernel regression^(18)^, Bayesian modeling algorithms^(19)^, and projection onto convex sets (POCSs)^(20)^. Most of these SR methods assume a priori knowledge about the motion model, blur kernel, and noise level. However, there are many cases where the actual image degradation process is unknown.

For this reason, many blind SR image reconstruction methods were developed. These methods usually involve two steps: (a) motion estimation for LR images, followed by (b) simultaneous estimation of both the HR image and the blurring function. Since separating image registration and HR estimation tends to produce sub-optimal results, some researchers have developed methods that jointly estimate motion parameters and the HR reconstruction^(21)^. Recently, similar to SISR problems, deep-learning-based methods have been proposed to simultaneously solve video SR and MISR problems. Most of the existing work is focused on video SR, such as frame recurrent SR^(22)^ which utilizes previous inferred HR frames to super-resolve subsequent frames in an end-to-end trainable framework that incorporates both frame registration and HR estimation. More recently, several deep-learning-based algorithms are proposed to solve MISR problems in satellite imaging and burst photography. HighRes-net^(23)^ learns to co-register, fuse, and upsample multiple frames into one super-resolved image in an end-to-end manner. Residual attention model (RAMS) utilizes 3D convolutions to exploit spatial and temporal relationships across images for HR reconstruction^(24)^ of satellite images. Deep burst SR^(25)^ combines both pixel-wise optical flow alignment and attention based fusion module to achieve HR reconstruction from image bursts.

Despite all the previous work of SR on natural and satellite images, little work has been done on MISR methods in the context of cryo-EM. Preliminary work done by Chen et al.^(26)^ demonstrated that MISR reconstruction surpassing the Nyquist frequency is possible by using a noiseless synthetic dataset and without considering the modulation effects of the contrast transfer function (CTF). Real micrographs acquired with an electron microscope, however, inevitably suffer from low-SNR due to the small doses used during imaging and are modulated by the CTF. Meanwhile, deep-learning techniques have been applied to cryo-EM imaging in a variety of other contexts, including particle picking^(27–30)^, automated micrograph and class selection^(31)^, CNNs for segmentation of cryo-electron tomograms^(32)^, map denoising and local resolution estimation^(33–35)^, and more recently the study of conformational heterogeneity during 3D reconstruction^(36)^. Inspired by the recent success of deep-learning-based MISR approaches, we aim to tackle the problem of SR using cryo-EM images by jointly registering LR images and reconstructing SR images, all within an end-to-end trainable network based on the ZSSR framework^(5)^. An early version of our approach was reported in Huang et al.^(37)^

## 3. Method

### 3.1. Deep internal statistics of cryo-EM micrographs

**Figure 2.**
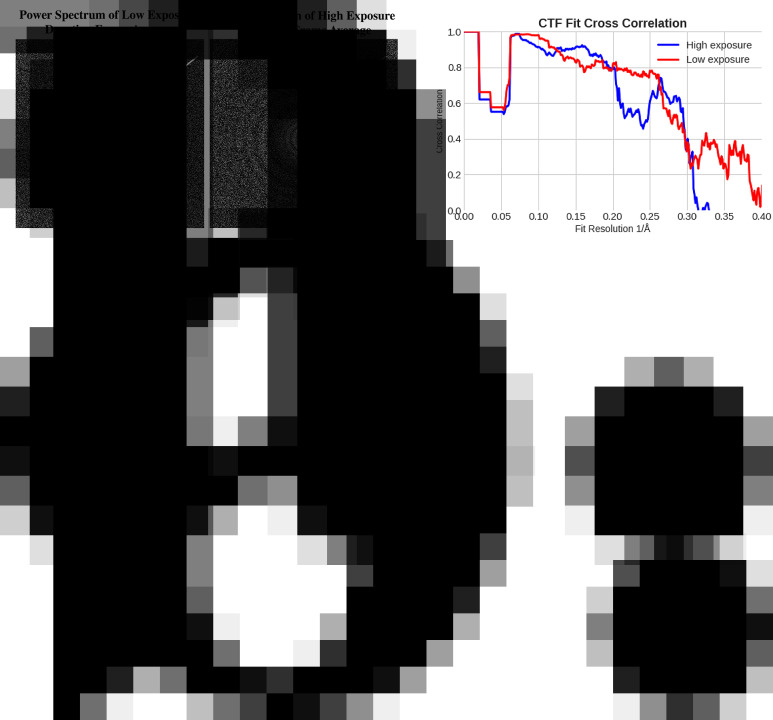
Internal predictive power of movie-specific information. (a) Power spectrum calculated from the average of the first half of frames (less radiation damage) and from the second half of frames (more radiation damage). As indicated by the white arrows, Thon rings are more visible in the first image which has less radiation damage compared to the second image that presents more radiation damage. As reported earlier, this shows that earlier frames in the exposure carry more high-frequency information than the later frames. (b) Cross-correlation between the fitted contrast transfer function (CTF) and the measured power spectrum. Similar to panel (a), the power spectrum computed from the early part of the exposure has higher cross-correlation compared with the theoretical CTF. The better cross-correlation fit confirms that the high-frequency signal is stronger in the first half of the exposure.

The key assumptions of our approach are that cryo-EM micrographs have data internal repetition and that due to radiation damage, the amount of signal available at different frequencies varies for each frame, as high-frequency information is degraded in frames with more radiation damage. Figure 1 shows an example of a single-particle cryo-EM micrograph (average from all raw movie frames). Since each micrograph contains hundreds of projections of the same protein-of-interest, data repetition occurs naturally. In addition, as data is collected in movie mode, early frames and late frames are subject to different radiation damage regimes. Earlier frames in the exposure contain more high-frequency information than later frames which are affected by radiation damage. As shown in Figure 2, at higher frequencies, Thon rings are more visible in micrographs obtained by averaging the first half of frames in the movie, compared to micrographs obtained by averaging the second half of the frames. This phenomenon was also empirically verified by Bartesaghi et al.^(38)^ and Grant and Grigorieff^(39)^ which compared 3D reconstructions of proteins obtained using frames from different exposure ranges, showing that frames from lower exposures achieve 3D reconstructions with higher resolutions compared to those obtained using later frames in the exposure.

### 3.2. Problem formulation

MISR aims at recovering an HR image 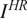 from a set of 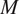 LR images 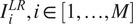 of the same scene acquired during a certain time window. Typically, a LR image 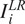 is related to the HR image 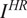 through motion shift, blurring, downsampling, and noise corruption:(1)

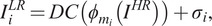

where 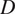 is the downsampling process, 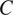 represents blurring, 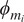 is the relative motion for each frame, and 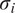 is the noise corruption. In the context of cryo-EM, 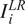 are the collected LR movie frames and 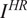 is the ground-truth HR image, without CTF modulation and free of noise. Motion shift mainly comes from beam-induced motion that occurs during data acquisition, blurring is modeled by the CTF, which describes how contrast (information) is transferred to the image in terms of the spatial frequency. Severe noise corruption is a result of low electron dosage during imaging. It is worth noting that the CTF, unlike other commonly used blurring kernels such as Gaussian, has multiple zero crossings, which means information at certain frequencies is completely lost, making direct inversion impossible. Denoising techniques applied at the single image-level are also prone to the removal of high-frequency information along with the actual noise. Therefore, in the standard cryo-EM data processing pipeline, CTF inversion and denoising is not applied until the final step of 3D reconstruction. In order to cope with the special characteristics of cryo-EM images described above, unlike most standard SR algorithms, instead of recovering an HR image 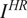 that is free of blurring and noise from LR images, we aim to generate 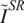 subject to the modulation by the CTF and noise using our proposed methodology 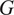 :(2)

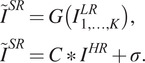



Since raw frames 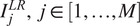 , where 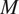 is the total number of raw frames in cryo-EM movies, have extremely low SNR and errors of SR reconstruction from LR images grow in proportion to the noise variance^(40)^, and frames collected under greater electron dosage have less high-frequency information due to radiation damage, we use moving averages of raw frames aligned to different reference frames (instead of using raw frames as LR inputs):(3)

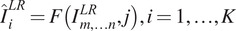

where 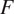 aligns and averages raw frames 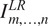 from the *m*th frame to the *n*th frame in the movie with respect to the reference frame 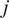 . Reference frame 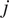 is selected at random from all possible frames 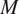 . These generated LR frame averages 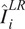 have higher SNR, and since each 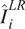 is aligned to a different reference frame, relative motions exist between these frame averages. Therefore, they are more suitable as LR inputs for SR reconstruction. In addition, as these inputs are obtained by averaging frames with high electron exposure, they contain limited high-frequency information.

### 3.3. Proposed framework

**Figure 3.**
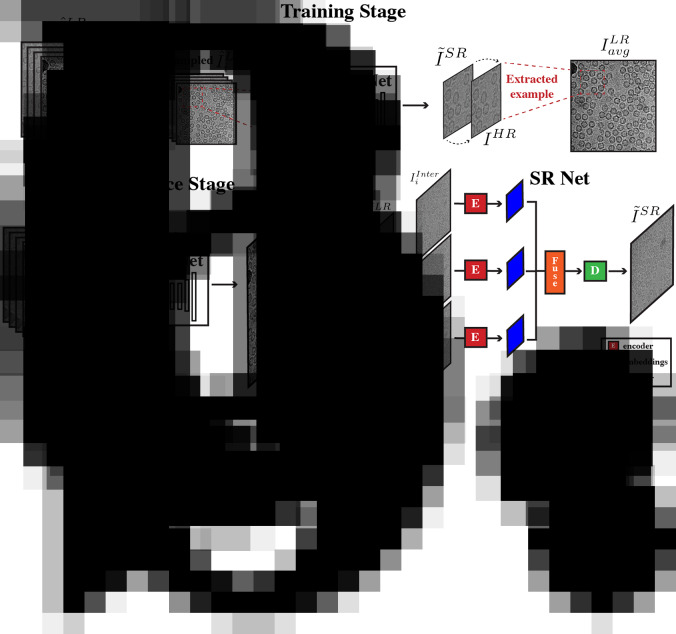
Overall cryo-ZSSR framework. (a) During the training stage, pseudo LR–HR pairs are formed using further downsampled frames that have more radiation damage (second half of frames in a movie) ( 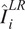 ) and averages of frames with less radiation damage ( 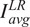 , first half of frames in a movie). Extracted patches of frames from further downsampled 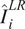 are fed into SR Net which produces a 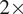 super-resolved image 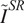 . SR Net learns to recover 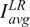 from the coarser input 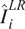 . (b) During the inference stage, the resulting self-supervised SR Net is then applied to the full 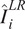 to produce its SR output. (c) Architecture of SR Net: input frames are first upsampled to the desired output size. The interpolated frames are used as inputs to SR Net. These frames are encoded, fused, and decoded to generate the final SR output.

Our proposed framework (Figure 3a,b) leverages both the strong internal predictive power and the generalization capabilities of deep neural networks. Given an input movie stack 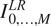 with 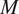 frames, we first divide the stack into two parts: (a) early frames in the exposure 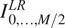 (first half of the frames), and (b) late frames in the exposure 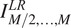 . We transform 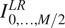 into 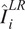 using Equation 3 and 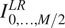 into 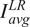 by aligning and averaging all frames in the low exposure stack. We further downsample 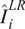 by a factor of 2 and treat the further downsampled frames as LR inputs to the network. We treat 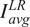 as a pseudo HR micrograph. The network learns to reconstruct 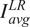 using further downsampled 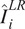 . Using this pseudo HR–LR pair, we are able to train our movie-specific SR Net without the need for any ground-truth HR images. To summarize, a movie-specific SR Net is trained in the following ways:

1. Extract example patches of fixed size from the input LR frames 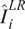 and the input pseudo HR image 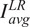 .
2. Further downsample the extracted examples from 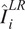 by a factor of 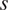 (we use 2). These downsampled examples now become temporary LRs.
3. Temporary HR–LR pair is formed using the downsampled extracted patches from 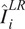 and its corresponding extracted patch from the pseudo HR image 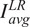 .
4. Feed temporary LR images obtained in step 2 into SR Net, a SR output is generated and compared with the temporary HR.

Once the network is trained, instead of using 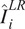 obtained using frames from the second half of the exposure, a new set of 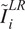 is formed by using averages of all raw frames aligned to different reference frames:(4)

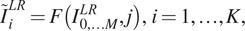

where *F* represents the application of a motion correction/alignment algorithm such as *MotionCor2*^(41)^. In this new setup, LR frames are used as input to the trained network and the desired SR output 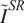 is constructed. By using 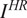 from the first half of the frames (that contain more high-frequency information) and learning to recover these high-frequency information from LR inputs, the movie-specific SR Net is able to leverage the power of cross-frequency internal recurrence of image-specific information. To further enrich the training dataset, data augmentation is applied to the set of LR images to extract more pairs of HR–LR to train on, including mirror reflections in the vertical and horizontal directions.

The overall architecture of SR Net is based on HighResNet^(23)^, which includes three main steps: encoding, fusion, and decoding (Figure 3c). The network learns to implicitly co-register multiple LR frames 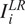 and fuse them into a single SR view. Unlike HighResNet, which upscales input LR views during the decoding step using a deconvolution layer, we first upscale LR inputs 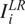 to the desired SR output size 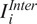 before feeding it into the encoder using bilinear interpolation. The network thus learns the residual between the interpolated LR and the HR images. A detailed description of the architecture of HighResNet is given in Deudon et al.^(23)^.

*Encode.* The encoding stage contains two steps: first, compute a reference micrograph and second, embed each frame jointly with the reference. The reference micrograph is computed as the median of all input LR frames 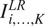 . The reference micrograph is concatenated to each frame and the concatenated reference-frame representations serve as inputs to the embedding layer. The shared reference micrograph serves as anchor for implicit alignment and encourages the network to learn differences across multiple frames.

*Fuse.* Encoded outputs 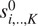 from the embedding layer are then fused recursively. At each time step 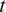 , after fusion, the number of encoded outputs is reduced by half. Given a pair of hidden states 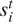 and 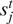 , the fusion step merges these two representations by first concatenating 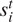 and 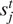 and then projecting to a new representation.

*Decode.* After 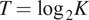 fusion steps, the final LR encoded representation 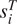 is fed into the decoder and the decoder outputs the final super-resolved micrograph, 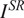 .

#### 3.3.1. Loss function

In addition to computing the mean absolute error (MAE) between the generated SR and the actual pseudo-HR images, we also use a Fourier domain frequency loss to help preserve CTF modulation.

*Fourier domain frequency loss.* For an image and its Fourier representation 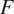 , denote 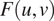 as the Fourier coefficient at spectrum coordinate 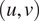 . Let 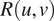 and 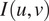 be its real and imaginary parts, we can rewrite 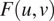 as(5)

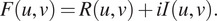



Let 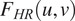 be the Fourier coefficient of the ground-truth HR image and 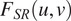 be the Fourier coefficient of the reconstructed SR image. Denote 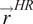 and 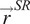 as respective vectors mapped from 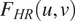 and 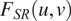 . By the definition of amplitude and phase of each Fourier coefficient, 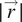 corresponds to the amplitude and 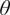 corresponds to the phase. Therefore, the frequency distance can be represented as the distance between 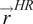 and 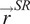 , which can be calculated using the 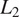 Euclidean distance:(6)

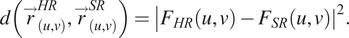



As both generated SR and pseudo-HR are corrupted by noise, this means that at higher frequency, its corresponding coefficients contain both information from underlying signal and noise. Therefore, minimizing distance at the high-frequency portion of the spectrum may lead to undesired learning of noise. To mitigate this effect, we reweight the loss at each frequency level using a Gaussian kernel. For higher frequencies, its resulting loss is down-weighted in the overall loss calculation. The final loss is calculated as the sum of the MAE loss and the weighted frequency loss.

#### 3.3.2. Implementation details

We use the ADAM optimizer, starting with a learning rate of .001 and we adaptively decrease the learning rate based on the training procedure proposed in Shocher et al.^(5)^. Training stops when the learning rate reaches 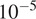 , at around 200 iterations. The network is trained to learn upscaling by a factor of 2. At each iteration, a fixed crop size of 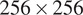 is used, while the 2× downsampled versions have size of 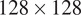 . This way, training time is independent of the input size. During the inference stage, the generated SR is further combined with the back-projection technique^(42,43)^. The final image is corrected by back-projection. Each set of LR images takes around 2 min to train for an upsampling factor of 2, the final SR image takes about 30 s to generate on an NVIDIA Tesla V100 GPU with 32GB of memory.

### 3.4. Overall data processing pipeline with cryo-ZSSR

As shown in Figure 1a, cryo-ZSSR serves as a pre-processing step to the overall cryo-EM 3D reconstruction process. To summarize, the new processing pipeline is as follows:

- For each movie stack 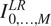 containing M frames, divide each stack into two parts: (a) early frames in the exposure 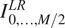 (first half of the frames), and (b) late frames in the exposure 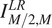 (second half of the frames).
- For 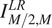 , align a subset of these frames with respect to different reference frames (we used four frames) using a motion correction algorithm such as MotionCorr to obtain 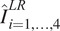 , Equation 3. For 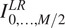 , align these frames with respect to the center frame to obtain 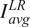 .
- Using 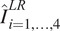 and 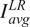 , generate pseudo HR–LR pairs and train the network by following steps in Section 3.3.
- Align all frames with respect to the reference frames used in the second step above and obtain a new set of 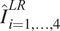 . Feed the new 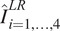 into trained network and obtain the final super resolved output 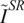 .
- Perform all the following data processing steps (CTF estimation, particle picking, orientation estimation, and 3D reconstruction) on 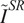 generated micrographs from each movie stack.

## 4. Experiments and Validation on Single-Particle Cryo-EM Data

To validate our approach, we used cryo-EM movies of apoferritin and the T20S proteasome from the Electron Microscopy Public Image Archive (EMPIAR) under accession codes 10146 and 10025^(3,44)^.

*EMPIAR-10146.* Apoferritin is a commonly used test sample that has a molecular weight of 440 kDa and octahedral symmetry (O). This dataset consists of 20 movies with 50 frames each and 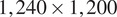 pixels in size. The physical pixel size is 1.5 Å and the images were acquired using a beam energy of 300 kV and an exposure rate of 2 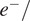 Å^2^ (equivalent to a total dose of 100 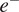 /Å^2^), Figure 1b. The original movies are subjected to the standard single-particle pipeline resulting in a 3.5 Å resolution reconstruction from 1,200 particles that was used as ground truth. We then binned the original movies by a factor of 2 using the IMOD program^(45)^ (resulting in a pixel size of 3 Å). Downsampled frames using uncropped frames had a size of 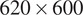 pixels and were aligned using MotionCorr2^(41)^ and averaged. The frames were aligned to four different reference frames and the corresponding frame averages were generated.

*EMPIAR-10025.* The T20S proteasome has a molecular weight of 750 kDa and D7 symmetry. We used a subset of this dataset which consists of 47 movies with 38 frames each and 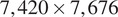 pixels in size. As the movies are acquired using microscope’s camera SR mode, the pixel size is 0.66 Å. The images were acquired using a beam energy of 300 kV and an exposure rate of 1.4 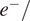 Å^2^ (equivalent to a total dose of 58 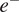 /Å^2^), Figure 1c. Instead of using the super-resolved movies as reference, we used movies binned by a factor of 2 (1.32 Å pixel size, 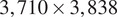 image size) as ground truth. LR movie frames are obtained by downsampling the original movies by a factor of 4 (2.64 Å pixel size, 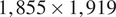 image size). We followed the same alignment and downsampling procedure as in EMPIAR-10406. For simplicity, we cropped sub-micrograph patches of size 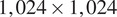 from the resulting LR frames and these sub-micrograph patches are used as the input LR images.

Movie-specific SR Nets were trained for each movie in both datasets. Once fully trained, LR images were upscaled by a factor of 2 through our framework. For EMPIAR-10406, the resulting SR image has size 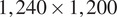 , which is the same as the original unbinned micrographs. For EMPIAR-10025, the resulting SR image has size 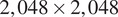 . The original HR reference movies were never used or seen during any part of the training or testing steps. The resulting SR micrographs now replaced the downsampled LR images and were used as inputs to the single-particle cryo-EM structure determination pipeline. The CTF of each SR micrograph was estimated using CTFFIND4^(46)^ and particles were extracted and subjected to iterative 3D refinement using the cisTEM package^(47)^. This process was repeated for the LR images, and the SR micrographs upsampled using bilinear interpolation and cryo-ZSSR. In both cases, we used the exact same particle stacks and estimated orientation parameters for 3D reconstruction.

### 4.1. Cryo-ZSSR improves the quality of individual micrographs

**Figure 4.**
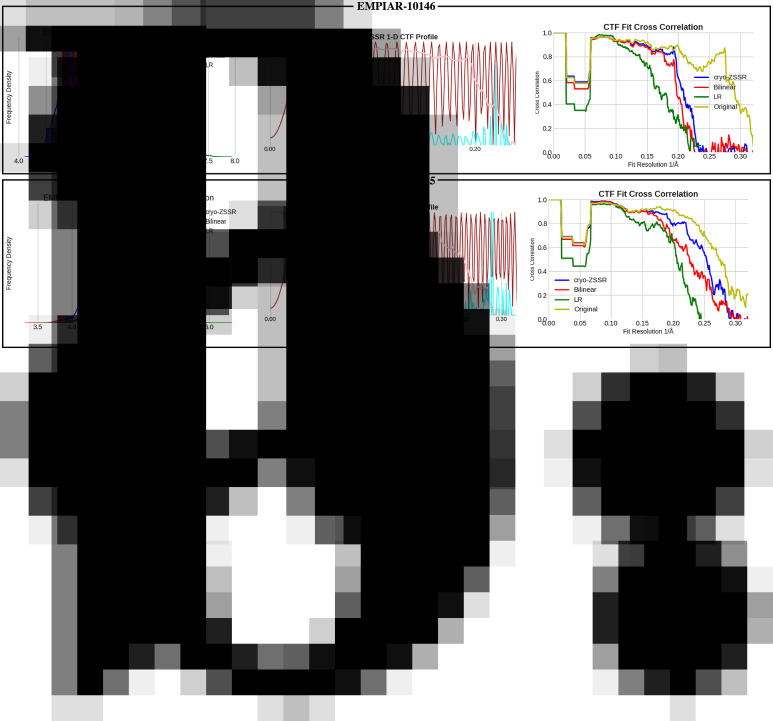
Cryo-ZSSR improves image quality metrics for individual micrographs. To evaluate the performance of cryo-ZSSR at the micrograph level, we estimated the CTF of movies in the EMPIAR-10146 and EMPIAR-10025 datasets before and after upsampling. (a) CTF statistics of EMPIAR-10146. Right: Histogram of estimated fit resolution showing the net improvement in image quality obtained by cryo-ZSSR (lower fit resolutions represent better results). Middle: Example 1D CTF radial profiles of cryo-ZSSR upsampled image. Left: Corresponding CTF Fit cross correlation score. As shown, the output from cryo-ZSSR has better cross correlation score compared to both bilinear interpolation and the low-resolution image. (b) CTF statistics of EMPIAR-10025. Similar to EMPIAR-10146, cryo-ZSSR is able to achieve better fit resolution, cross correlation score compared to LR input and the bilinear interpolated image. Right: Histogram of estimated fit resolution. Middle: Example 1D CTF radial profiles of cryo-ZSSR upsampled images. Left: Corresponding CTF fit cross correlation score for LR, bilinear interpolation, cryo-ZSSR and original inputs.

To test the performance of our algorithm on individual micrographs, we estimated the CTF of each of the micrographs in both datasets using three sets of images (LR, upsampled using bilinear interpolation, and upsampled using cryo-ZSSR) and the ground-truth original image. Specifically, we quantified the overall improvement in image quality by measuring the estimated fit resolution for images in both datasets. Estimated fit resolution gives an indication of how far the signal extends (lower numbers are better). As shown in Figure 4, left, in both datasets, SR images reconstructed using our approach have better fit resolution than both the LR and bilinear interpolated images, indicating that cryo-ZSSR can effectively recover HR information present in the LR movie stacks. In addition, we show 1D CTF radial profiles of a representative cryo-ZSSR upsampled image and its corresponding CTF cross correlation fit compared to LR, bilinear interpolated and original images (Figure 4, right). As shown, the cross-correlation fit results indicate that the strength of the signal present in the cryo-ZSSR result is higher when compared to the LR and bilinear interpolated images, even though it is not as good as the original input. This implies that although cryo-ZSSR can recover some high-frequency information present in the LR movie stacks, it cannot achieve perfect recovery. In addition, some high-frequency information is permanently lost during the downsampling process, making full recovery impossible.

### 4.2. Cryo-ZSSR improves the resolution of 3D reconstructions

**Figure 5.**
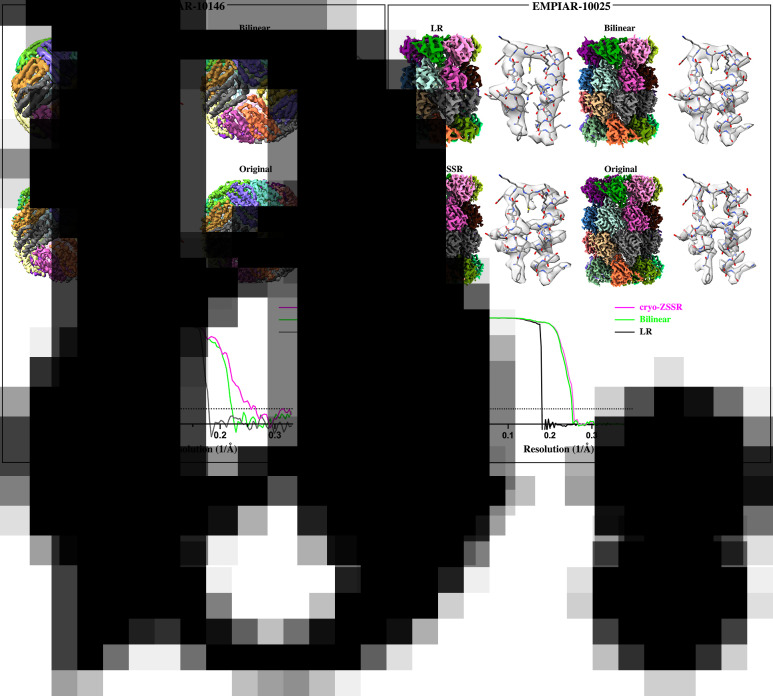
Cryo-ZSSR upsampled images improve the resolution of 3D structures. To evaluate the performance of cryo-ZSSR at the 3D level, we performed 3D reconstruction for both apoferritin (EMPIAR-10146) and T20S proteasome (EMPIAR-10025) datasets. In each case, reconstructions were obtained using the same set of particles. (a) Overall structure of apoferritin and zoomed-in view of an alpha helix with fitted atomic model, for maps obtained from the LR images (top left), upsampled images using bilinear interpolation (top right), upsampled images using cryo-ZSSR (bottom left), and ground-truth images (bottom right). Fourier shell correlation (FSC) curves for maps obtained using LR images (gray), upsampled using bilinear interpolation (green), and upsampled using cryo-ZSSR (magenta) against ground-truth reconstruction (bottom). Estimated resolutions are 6.0 Å, 4.8 Å, and 3.9 Å, respectively, based on the 0.143-cutoff (dotted line). Lower numbers represent better reconstruction quality. (b) Overall structure of T20S proteasome and zoomed-in view with fitted atomic model. Similar to EMPIAR-10146, cryo-ZSSR is able to achieve better 3D resolution. FSC curves for maps obtained using LR images (gray), upsampled using bilinear interpolation (green), and upsampled using cryo-ZSSR (magenta) against ground-truth reconstruction (bottom). Estimated resolutions are 3.9 Å, 4.0 Å, and 5.5 Å, respectively, based on the 0.143-cutoff (dotted line). Due to low sampling rate, the FSC for the LR reconstruction has a rapid decay at 5.5 Å.

We also evaluated the downstream effects of our SR interpolation algorithm by measuring the quality of the final 3D reconstructions. The three sets of movies (LR, upsampled using bilinear interpolation, and cryo-ZSSR) were used as input to the standard single-particle refinement pipeline implemented in cisTEM. For EMPIAR-10406, 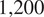 particles were selected and aligned against an external reference of apoferritin using iterative projection matching. We repeated this process using the three sets of images and the ground-truth data (Figure 5). Consistent with the CTF estimation results, the features and resolution of the cryo-ZSSR map are better than the ones obtained using bilinear interpolation and the LR data, with estimated resolutions according to the 0.143-FSC criteria of 3.9 Å, 4.8 Å, and 6.0 Å, respectively (Figure 5a). The resolution obtained using the ground-truth images is 3.5 Å. Lower numbers indicate better reconstruction quality. The resolution obtained by cryo-ZSSR clearly surpasses the 6 Å Nyquist limit imposed by the original physical pixel size of 3 Å, and the reconstruction shows clear density for side chains, in agreement with the atomic model and corresponding structural features in the ground-truth map. For EMPIAR-10025, 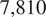 particles were selected as inputs to ab-initio reconstruction and homogeneous refinement. Similarly, the features and resolution of the cryo-ZSSR map are better than the ones obtained using bilinear interpolation and LR images, with estimated resolutions according to the 0.143-FSC criteria of 3.9 Å, 4.0 Å, and 5.5 Å (Figure 5b). The resolution obtained using the ground-truth images was 3.1 Å. While our proposed method is able to obtain better reconstruction compared to bilinear interpolation, the improvement is not as significant as compared to EMPIAR-10146. It should be noted that for LR images with a pixel size of 2.64 Å, the Nyquist limit is 5.28 Å. However, the current reconstruction method is only able to obtain a resolution of 5.53 Å, indicating that the resolution is limited by other factors (less symmetry) in addition to the pixel size. This reveals a potential limitation of our proposed method: when there is less symmetry, which leads to less internal data repetition, even though it is still able to outperform traditional interpolation-based upsampling methods, the degree of improvement is less significant.

## 5. Discussion and Conclusion

We present a neural network framework to upsample low-SNR single-particle cryo-EM movies using a MISR algorithm based on self-supervised deep internal learning. By leveraging information repetition across multiple frequencies in collected cryo-EM movies, we are able to train the network without the need of ground-truth or prior training using HR images. Applications of this technique to a LR dataset of apoferritin sampled at 3 Å/pixel resulted in a three-dimensional reconstruction at 3.9 Å resolution where side chains could be visualized at a similar level of detail seen in the ground-truth map. On a LR dataset of T20S proteasome sampled at 2.64 Å/pixel, after SR upsampling, a three-dimensional reconstruction at 3.9 Å resolution is obtained. These experiments suggest that cryo-ZSSR is an effective strategy to recover HR information contained in low-SNR, LR cryo-EM movies. The proposed framework is most suitable for datasets with relatively high symmetry that achieve the Nyquist frequency during 3D reconstruction, where the main limitation factor is the pixel size. For datasets where the resolution is limited by factors other than the pixel size, the resolution improvements brought about by our method will be less significant. Admittedly, our proposed algorithm has some limitations: first, it does not have an explicit motion compensation component. While implicit registration already results in SR images that contains more high-frequency information, accurate sub-pixel motion estimation is key in further improving the quality of the SR image. Second, the algorithm does not presently account for the resolution-lowering effects caused by radiation damage affecting cryo-EM samples, as current fusion and decoding steps do not incorporate dose weighting. Previous research shows that by accounting for radiation damage through dose weighting, the resolution of 3D reconstructions can be improved. Therefore, incorporating frequency-domain Fourier coefficient reweighting has the potential of obtaining further improvements in resolution. As cryo-EM datasets typically contain thousands of micrographs, training of our image-specific network can take a long time. To this end, we are investigating the possibility of training a dataset-specific network. In addition, we will continue investigating how protein symmetry can affect the performance of the algorithm, as symmetry plays an important role in internal statistics recurrence. Overall, the proposed SR strategy may be used in conjunction with lower magnification imaging to accelerate data collection without sacrificing image quality.

## Data Availability

Replication data and code can be found in https://gitlab.cs.duke.edu/bartesaghilab/cryo-zssr.
